# Feasibility, Yield, and Cost of Active Tuberculosis Case Finding Linked to a Mobile HIV Service in Cape Town, South Africa: A Cross-sectional Study

**DOI:** 10.1371/journal.pmed.1001281

**Published:** 2012-08-07

**Authors:** Katharina Kranzer, Stephen D. Lawn, Gesine Meyer-Rath, Anna Vassall, Eudoxia Raditlhalo, Darshini Govindasamy, Nienke van Schaik, Robin Wood, Linda-Gail Bekker

**Affiliations:** 1 Department of Clinical Research, Faculty of Infectious and Tropical Diseases, London School of Hygiene and Tropical Medicine, London, United Kingdom; 2 The Desmond Tutu HIV Centre, Institute for Infectious Disease and Molecular Medicine, Faculty of Health Science, University of Cape Town, South Africa; 3 Department of Global Health and Development, London School of Hygiene and Tropical Medicine, London, United Kingdom; 4 Health Economics and Epidemiology Research Office (HE2RO), Department of Medicine, Faculty of Health Sciences, University of the Witwatersrand, Johannesburg, South Africa; 5 Center for Global Health and Development, Boston University, Boston, United States of America; 6 Department of Development Policy and Practice, Royal Tropical Institute, Amsterdam, The Netherlands; McGill University, Canada

## Abstract

**Background:**

The World Health Organization is currently developing guidelines on screening for tuberculosis disease to inform national screening strategies. This process is complicated by significant gaps in knowledge regarding mass screening. This study aimed to assess feasibility, uptake, yield, treatment outcomes, and costs of adding an active tuberculosis case-finding program to an existing mobile HIV testing service.

**Methods and Findings:**

The study was conducted at a mobile HIV testing service operating in deprived communities in Cape Town, South Africa. All HIV-negative individuals with symptoms suggestive of tuberculosis, and all HIV-positive individuals regardless of symptoms were eligible for participation and referred for sputum induction. Samples were examined by microscopy and culture. Active tuberculosis case finding was conducted on 181 days at 58 different sites. Of the 6,309 adults who accessed the mobile clinic, 1,385 were eligible and 1,130 (81.6%) were enrolled. The prevalence of smear-positive tuberculosis was 2.2% (95% CI 1.1–4.0), 3.3% (95% CI 1.4–6.4), and 0.4% (95% CI 1.4 015–6.4) in HIV-negative individuals, individuals newly diagnosed with HIV, and known HIV, respectively. The corresponding prevalence of culture-positive tuberculosis was 5.3% (95% CI 3.5–7.7), 7.4% (95% CI 4.5–11.5), 4.3% (95% CI 2.3–7.4), respectively. Of the 56 new tuberculosis cases detected, 42 started tuberculosis treatment and 34 (81.0%) completed treatment. The cost of the intervention was US$1,117 per tuberculosis case detected and US$2,458 per tuberculosis case cured. The generalisability of the study is limited to similar settings with comparable levels of deprivation and TB and HIV prevalence.

**Conclusions:**

Mobile active tuberculosis case finding in deprived populations with a high burden of HIV and tuberculosis is feasible, has a high uptake, yield, and treatment success. Further work is now required to examine cost-effectiveness and affordability and whether and how the same results may be achieved at scale.

## Abstract 2

Katharina Kranzer and colleagues investigate the operational characteristics of an active tuberculosis case-finding service linked to a mobile HIV testing unit that operates in underserviced areas in Cape Town, South Africa.

## Editors' Summary

**Background:**

In 2010, 8.8 million people developed active tuberculosis—a contagious bacterial infection—and 1.4 million people died from the disease. Most of these deaths were in low- and middle-income countries and a quarter were in HIV-positive individuals—people who are infected with HIV, the virus that causes AIDS, are particularly susceptible to tuberculosis because of their weakened immune system. Tuberculosis is caused by *Mycobacterium tuberculosis*, which is spread in airborne droplets when people with the disease cough or sneeze. Its characteristic symptoms are a persistent cough, unintentional weight loss, hemoptysis (coughing up blood from the lungs), fever, and night sweats. Diagnostic tests for tuberculosis include sputum smear microscopy (microscopic analysis of mucus brought up from the lungs by coughing) and culture (growth) of *M. tuberculosis* from sputum samples. Tuberculosis can be cured by taking several powerful antibiotics daily for at least 6 months.

**Why Was This Study Done?:**

To improve tuberculosis control, active disease must be diagnosed quickly and treated immediately. Passive tuberculosis case finding, which relies on people seeking medical help because they feel unwell, delays the diagnosis and treatment of tuberculosis and increases *M. tuberculosis* transmission. By contrast, active tuberculosis case finding—where health workers seek out and diagnose individuals with TB who have not sought care on their own initiative—has the potential to reduce tuberculosis transmission by improving case detection. The World Health Organization (WHO), which already recommends active tuberculosis case finding in HIV-infected individuals as part of its HIV/TB “Three I's” strategy, is currently developing guidelines to inform the design of national tuberculosis screening strategies based on the local prevalence of HIV and TB and other context-specific factors that affect how many individuals need to be screened to identify each additional new tuberculosis case (the “yield” of active case finding). Large gaps in our knowledge about mass-screening strategies are complicating the development of these guidelines so, in this observational prospective study, the researchers assess the feasibility, uptake, yield, treatment outcomes, and costs of adding an active tuberculosis case-finding program to an existing mobile HIV testing service in South Africa.

**What Did the Researchers Do and Find?:**

All HIVnegative adults with symptoms characteristic of tuberculosis and all HIV-positive adults regardless of symptoms who attended a mobile HIV testing service operating in deprived communities in ape Town, South Africa between May 2009 and February 2011 were eligible for inclusion in the study. Of the 6,309 adults who accessed the mobile clinic during this period, 1,385 met these eligibility criteria, and 1,130 were enrolled and referred for the collection of sputum samples, which were analyzed by microscopy and culture. The prevalence of smear-positive tuberculosis was 2.2%, 3.3%, and 0.4% among HIV-negative study participants, newly diagnosed HIV-positive participants, and people already known to have HIV, respectively. The corresponding prevalences for smear-negative/culture-positive tuberculosis were 5.3%, 7.4%, and 4.3%, respectively (culture detects more tuberculosis cases than microscopy but, whereas microscopy can provide a result within 1–2 days, culture can take several weeks). Fifty-six new tuberculosis cases were identified, 42 people started tuberculosis treatment, and 34 completed treatment (a treatment success rate of 81%). Finally, the incremental cost of the intervention was US$1,117 per tuberculosis case detected and US$2,458 per tuberculosis case cured.

**What Do These Findings Mean?:**

These findings show that active case finding for tuberculosis delivered through a mobile HIV testing service is feasible and has a high uptake, yield and treatment success in deprived communities with a high prevalence of HIV and tuberculosis. The findings also highlight the challenges faced by mobile population-based services such as losses between tuberculosis diagnosis and treatment, which were greatest for smear-negative/culture-positive people who were more difficult to contact than smear-positive people because of the greater time lag between sputum collection and diagnosis. Because the study was done in a single city, these findings need to be confirmed in other settings—the yield of active tuberculosis case finding reported here, for example, is not likely to be generalizable to countries that rely on sputum smears for tuberculosis diagnosis. Finally, given that the incremental cost per case treated in this study is 3-fold higher than the incremental cost per case treated under passive case detection in South Africa, further studies are needed to determine the cost-effectiveness and affordability of population-based tuberculosis screening.

**Additional Information:**

Please access these Web sites via the online version of this summary at http://dx.doi.org/10.1371/journal.pmed.1001281.

- The World Health Organization provides information on all aspects of tuberculosis, including information on tuberculosis and HIV, and on the Three I?s for HIV/TB (some information is in several languages); details of a 2011 meeting on the development of guidelines on screening for active tuberculosis are available
- The Stop TB partnership is working towards tuberculosis elimination; patient stories about tuberculosis/HIV coinfection are available
- The US Centers for Disease Control and Prevention has information about tuberculosis, about tuberculosis and HIV co-infection, and about the diagnosis of tuberculosis disease
- The US National Institute of Allergy and Infectious Diseases also has detailed information on all aspects of tuberculosis
- MedlinePlus has links to further information about tuberculosis (in English and Spanish)
- The Tuberculosis Survival Project, which aims to raise awareness of tuberculosis and provide support for people with tuberculosis, provides personal stories about treatment for tuberculosis; the Tuberculosis Vaccine Initiative also provides personal stories about dealing with tuberculosis

## Introduction

Active tuberculosis (TB) case finding in HIV-infected individuals has been recommended by the World Health Organization (WHO) as part of the “Three I's” policy initiative [1],[2]. Screening of household contacts of infectious TB cases has been recommended [3]–[5], but population-wide mass screening has been widely discouraged because of high cost and poor sustainability [6]–[8]. However, a recent study from Zimbabwe reported a decline of TB prevalence from 6.5 to 3.7 per 1,000 adults associated with community-level active TB case finding [6], leading to renewed interest in population-wide interventions.

Active TB case finding aims to reduce barriers for early TB case detection, including delays in presentation to health facilities, identification of the person as a TB suspect, and initiation of appropriate investigations. The ultimate goal of active TB case finding is to reduce TB transmission in the community through improved case detection and reduction in diagnostic delays [8].

The yield of active TB case finding defines the number of individuals who need to be screened to identify each additional new case. This yield is context specific, depending on TB and HIV prevalence, TB control services, and specificity and sensitivity of the screening tool used [9]. Other important parameters framing decisions on implementing active TB case finding include the feasibility and cost of screening, laboratory capacity, diagnostic tests available, and treatment outcomes in newly detected cases.

The WHO is currently developing guidelines on screening for TB disease to inform national TB screening strategies on the basis of the local epidemiological, demographic, and health system situation [7]. However, the development of these guidelines is complicated by significant gaps in knowledge regarding mass screening strategies in high TB and HIV prevalence settings. In particular, the screening strategy, optimal choice of diagnostic test, the cost of active TB case finding, and the treatment outcomes of actively detected cases remain unclear.

We report the results of a study investigating the feasibility, uptake, treatment outcomes, and cost of adding an active TB case-finding program linked to an existing mobile HIV testing service in Cape Town, South Africa.

## Methods

### Setting

This study was conducted at a mobile HIV testing service over 19 mo from May 2009 to February 2011. The service operated in underserviced peri-urban areas in greater Cape Town, South Africa [10]. This nurse-run and counsellor-supported mobile unit provided free HIV counselling and testing in combination with free screening for other chronic conditions (hypertension, diabetes, and obesity) and TB. Rapid HIV testing was performed according to the guidelines of the Provincial Government of the Western Cape [11]. The mobile unit was parked at township shopping centres, taxi ranks, stations, and the road side. The service was not formally advertised. However, the mobile van was painted in vibrant colours that therefore attracted a lot of attention. Most locations were served at least on a 3-monthly basis to allow individuals repeat testing.

### Mobile Clinic Procedures

**Figure 1:**
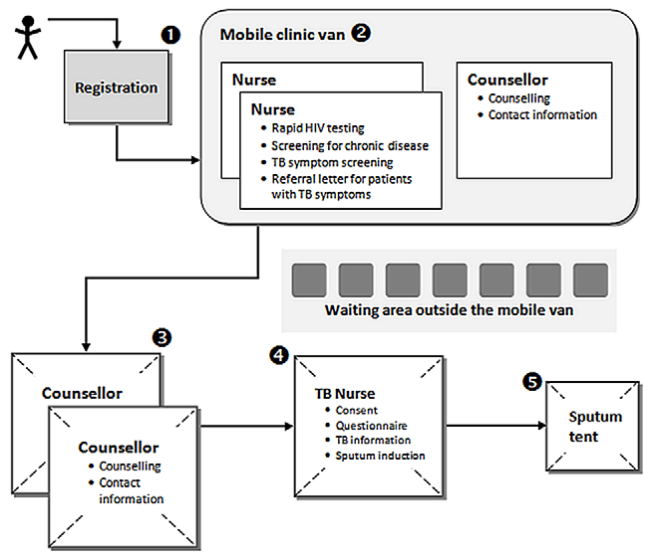
Procedures and patient flow in the mobile clinic. The number indicate the squence of procedure each patient had to go through.

All individuals accessing the mobile clinic were registered using a biometric fingerprint system, with no names or other personal identifiers collected in the registration process (Figure 1). They were then seen by a nurse for rapid HIV testing and chronic disease screening, including TB symptom screening, followed by a one-on-one counselling session. All individuals with conditions needing follow-up such as newly diagnosed hypertension, diabetes, HIV, or symptoms suggestive of TB (TB suspects) received a referral letter to take to their nearest clinic for further evaluation. Symptoms suggestive of TB were defined as cough for more than 2 wk, unintentional weight loss, haemoptysis, fever, and night sweats.

### Study Procedures

#### Screening

All HIV-negative adults with symptoms suggestive of TB and all adults with HIV infection or diabetes regardless of symptoms were eligible for the study. The lay counsellor collected detailed contact information from these individuals and referred them to the study nurse for participation in the study (Figure 1). A questionnaire was administered to all consenting individuals. They were asked to provide one sputum sample and were encouraged to do so by sputum induction, but could choose to provide a spot sample. Sputum samples were refrigerated overnight and sent to the laboratory the following day. The samples underwent fluorescence microscopy and liquid culture.

#### Sputum induction

Individuals were asked to rinse their mouth with water before the procedure. Sputum induction was performed outdoors in an open-roofed tent using 1.5–2-ml sterile 3% hypertonic saline solution and an ultrasonic Flo-Eolo nebuliser (CA-Mi) powered by a generator. Individuals breathed through the nebuliser mouthpiece until a satisfactory sample was produced.

#### Laboratory procedure

Sputum samples were analysed by accredited public-sector laboratories. Following decontamination with *N*-acetyl-*L*-cysteine sodium hydroxide, centrifuged sputum deposits were examined for acid-fast bacilli using auramine-O-fluorescent stain and cultured using mycobacterial growth indicator tubes (MGIT, Becton-Dickinson). Bacillary density was graded as scanty, 1+, 2+, and 3+, and all such smears were defined as “smear-positive.” The time to automated culture growth detection was recorded. Culture isolates positive for acid-fast bacilli were identified as *Mycobacterium tuberculosis* or as Mycobacterium other than tuberculosis (MOTT) complex and assessed for genotypic resistance using the MTBDRplus assay (Hain Lifesciences). Isolates also underwent phenotypic resistance testing for rifampicin and isoniazid by automated liquid MGIT culture.

#### Definitions

Symptom screen positive was defined as any of the following: cough >2 wk, weight loss, fever, night sweats, or haemoptysis. Patients with *M. tuberculosis* cultured from the sputum sample were defined as cases of TB. TB treatment outcomes were defined as cured, treatment completed, died, defaulted, and treatment interruption.

#### Follow-up

TB results were received from the laboratory on a daily basis by post and email. The laboratory contacted the research nurse by phone with smear-positive results and by email with culture-positive results, who then contacted individuals to inform them of their positive result and refer them to a TB clinic of their choice. If necessary, more than one attempt was made to contact individuals either by phone, home visit, or letter. The patient was given a referral letter or the referral letter was sent and faxed to the nearest TB clinic. Clinics were contacted by the research nurse to ascertain each patient's date of TB treatment initiation, TB register number, treatment outcomes, and date of outcome. The research nurse performed clinic visits to validate the information and check the TB register. Individuals who did not attend the clinic were contacted again to encourage linkage to care.

### Cost Analysis

An incremental cost analysis investigating the cost of adding TB screening using sputum induction to the existing mobile HIV testing service was performed adopting a health service provider perspective. Financial costs included the costs of human resources (clinical nurse practitioner, programme manager, counsellor), equipment, consumables, transport, laboratory tests, and office rent and overhead. Costs were divided into capital and recurrent costs [12], and capital costs were annualized and discounted at 6% per year [13]. Cost data from previous years were adjusted for inflation to 2011 constant costs [14] and converted to US$ (US$1.00 = ZAR 7.40) [15]. Some of the resources such as staff and space were jointly used with other services and were allocated proportionally. In order to analyse what staff time had been spent exclusively on TB screening activities, time-motion studies of all staff involved were conducted over 1 wk in August 2010 and 2 wk in January 2011, with a total of 13 complete screening days being observed. The two time periods were chosen because of seasonal variations in attendance rates and working conditions. Details of costs, proportional allocation, and time spent on screening are presented in Tables S1 and S2. Additionally, the research nurse kept a log file to estimate staff time spent on follow-up of individuals with positive smears and/or cultures. The additional diagnostic costs outside the mobile clinic were included. All individuals were asked about additional diagnostic tests such as chest X-rays, smears, and cultures performed in the primary health care clinic, which were then taken into account. Cost of first-line TB treatment was obtained from the literature [16],[17]. For patients who died or defaulted treatment, costs were allocated proportionately on the basis of their time spent on treatment.

Sensitivity analyses were conducted for different levels of staff salaries and 0% and 3% discount rates for capital costs, assuming that the outcomes would remain the same. The study was conducted by a clinical nurse practitioner (research nurse) and a doctor (program manager). A clinical nurse practitioner is a registered nurse with additional training to enable her to do a physical examination and prescribe medication. The sensitivity analysis was conducted substituting the clinical nurse practitioner by a staff nurse or a lay counsellor. A staff nurse is a nurse with 2 y training. A lay counsellor undergoes 6 wk of disease-specific training.

#### Measure of effectiveness

Effectiveness was measured as the prevalence of smear- and/or culture-positive disease detected, and the proportion of individuals with TB disease with a positive treatment outcome (cured or treatment completed).

### Statistical Analysis and Sample Size

Sample size calculations were based on precision. Pilot data showed that HIV-infected clients accessing the mobile service were less immuno-deficient than clients accessing stationary services [10]. Therefore a lower yield of TB screening was assumed in these individuals compared to the median yield of 8.5% in stationary services [9]. Sample size calculation assumed an overall yield of 2.5% (precision of ±1%) and 5% (precision of ±1.5%). Estimated sample sizes were 937 and 811. 1,200 individuals were predicted to be eligible over a 20-mo recruitment period (TB screening: 2 d per week, seven eligible individuals per day), and 20% of them were assumed to not undergo screening.

All analyses were carried out using Stata version 11.0 (Stata Corp. LP). Proportions were calculated for categorical variables, and medians and interquartile ranges (IQR) for continuous variables. The mean cost per examined sputum and TB case identified was calculated by summing the cost of all resources used for screening and dividing them by the number of sputum samples and TB cases diagnosed. The mean incremental cost per TB case with positive treatment outcome was calculated by summing the cost of all resources including treatment costs and dividing them by the number of TB cases with positive outcomes.

## Results

### Operational Data

**Figure 2:**
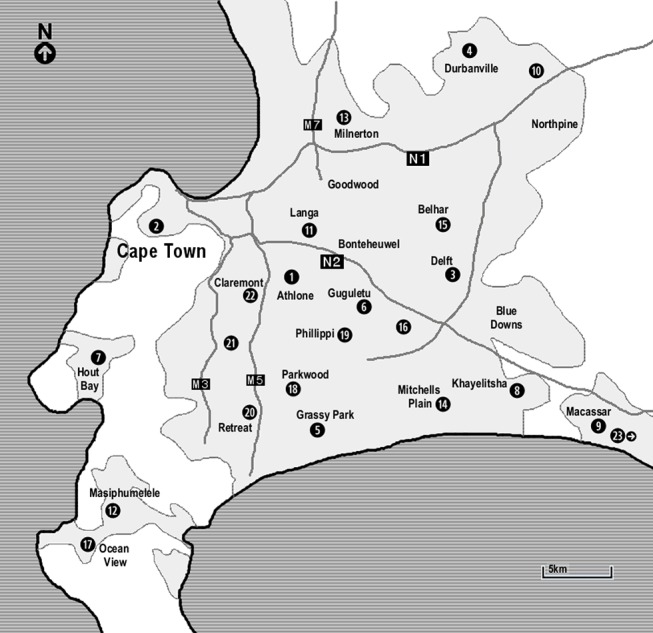
Map of Cape Town indicating the main areas in which the mobile services operated. (1) Athlone: testing at a shopping mall/market, the roadside, a social housing project; (2) Cape Town city bowel: testing at a college, service for homeless, service for commercial sex workers, two companies, two road sides; (3) Delft: testing at two squatter camps, two clinics, two social housing projects, the road side; (4) Durbanville: testing at two taxi ranks; (5) Grassy Park: testing at the road side; (6) Guguletu: testing at two shopping malls/markets, a clinic; (7) Hout Bay: testing at a school, in a township, at the harbour; (8) Khayelitsha: testing at a shopping centre/market, a school, in the township, at the station; (9) Macassar: testing at the road side; (10) Kraaifontain: testing at a clinic; (11) Langa: testing at a shopping mall/market, the road side; (12) Masiphumelele: testing in the township, at a shopping mall; (13) Milnerton: testing at a company; (14) Mitchells Plain: testing at the road side, a social housing project; (15) Belhar: testing at a squatter camp; (16) Nyanga: testing at a taxi rank, at a shopping centre; (17) Ocean View: testing at a clinic, in the township; (18) Parkwood: testing at two road sides; (19) Phillippi: testing at two farms, three road sides; (20) Retreat: testing at a clinic; (21) Wynberg: testing at the road side; (22) Claremont: testing at the road side; (23) Grabouw: testing in the township, at the clinic, at the road side.

TB screening was performed on 181 d over a period of 19 mo at 58 different sites. The majority of sites were in deprived areas, near townships and squatter camps (Figure 2). A total of 6,394 adults accessed the services of the mobile clinic: 85 were not tested for HIV, 5,551 tested HIV negative, 370 were newly diagnosed with HIV, and 388 were known HIV positive. Overall HIV prevalence in individuals tested was 11.9%. The median number of adults tested for HIV per day was 34 (IQR 27–41). The median number of individuals screened for TB was 6 (IQR 3–8), with a maximum number of 23 individuals per screening day.

### Uptake of TB Screening

**Figure 3:**
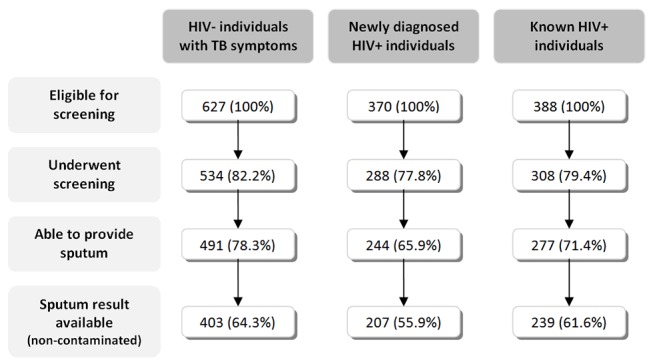
Flowchart of individuals participating in the study.

A total of 1,385 individuals (21.7% of all adults accessing the service) were eligible for TB screening through sputum induction: 627 were HIV negative, 370 were newly diagnosed HIV positive, and 388 were known HIV positive (Figure 3). 1,130 (81.6%) of all eligible individuals underwent screening. Individuals who were not screened were younger, more likely to be HIV positive, and had a higher body mass index (BMI) compared to individuals who underwent TB screening.

Of the 255 individuals who failed to undergo screening, 164 individuals did not want to wait or left without seeing the TB nurse, and 91 individuals were missed because the nurses or counsellors did not refer them for screening.

Of the eligible individuals 118 individuals were unable to produce a sputum sample, and 164 individuals had a contaminated sputum result. Overall, 60.9% of all eligible individuals had an interpretable sputum result (Figure 3).

### Baseline Characteristics

The median age of patients undergoing screening was 35.9 y, and 35.9% were men (Table 1). The majority of individuals lived in informal settlements or squatter camps (63.1%) and had less than 12 y of school education (78.0%), the minimum requirement to enter tertiary education. Financial insecurity was high with 22.7% of individuals reporting no regular income. Among individuals with regular income, only 35.3% were in employment or regular work. The median income was ZAR 1,000 (equivalent to US$135) per month. The national poverty line in 2008 was ZAR 515 per month and the average minimum wage for 2011 ZAR 1,500 per month.

**Table 1:** Socio-demographic and clinical characteristics and health-seeking behaviour among those undergoing screening.

Variables	Total (*n* = 1,130)	HIV− (*n* = 534)	Newly Diagnosed HIV+ (*n* = 288)	Known HIV+ (*n* = 239)
**Socio-demographic (** ***n*** ** = 1,130)**				
Median (IQR) **age (y)**	35.9 (27.9–45.7)	40.2 (29.2–50.0)	33.4 (26.4–40.9)	33.5 (28.2–40.8)
*n* (%) **male gender**	406 (35.9%)	261 (48.9%)	96 (33.3%)	49 (15.9%)
***Smoking*** a				
*n* (%) **never**	656 (58.2%)	224 (42.0%)	197 (68.6%)	235 (76.6%)
*n* (%) **stopped**	13 (1.2%)	8 (1.5%)	2 (0.7%)	3 (1.0%)
*n* (%) **currently**	458 (40.6%)	301 (56.5%)	88 (30.7%)	69 (22.5%)
***Alcohol consumption*** b				
*n* (%) **never**	644 (57.2%)	280 (52.7%0	160 (55.6%)	204 (66.5%)
*n* (%) **once per week**	229 (20.3%)	98 (18.5%)	67 (23.3%)	64 (20.9%)
*n* (%) **2–3 times per week**	190 (16.9%)	108 (20.3%)	52 (18.1%)	30 (9.8%)
*n* (%) **every day**	63 (5.6%)	45 (8.5%)	9 (3.1%)	9 (2.9%)
***Current relationship*** c				
*n* (%) **single**	677 (60.0%)	296 (55.4%)	184 (63.9%)	197 (64.4%)
*n* (%) **partner**	377 (33.4%)	199 (37.3%)	85 (29.5%)	93 (30.4%)
*n* (%) **divorced**	22 (2.0%)	15 (2.8%)	4 (1.4%)	3 (1.0%)
*n* (%) **widowed**	52 (4.6%)	24 (4.5%)	15 (5.25)	13 (4.3%)
*n* (%) **regular income**	985 (87.3%)	472 (88.2%)	239 (83.0%)	275 (89.6%)
***Source of income***				
*n* (%) **government grants**	259 (26.5%)	121 (26.0%)	40 (16.7%)	98 (35.8%)
*n* (%) **casual work**	375 (38.3%)	184 (39.5%)	96 (40.2%)	95 (34.7%)
*n* (%) **regular work**	345 (35.3%)	161 (34.6%)	103 (43.1%)	81 (29.6%)
Median (IQR) **income per month (ZAR)**	1,000 (400–1,200)	1,000 (400–1,200)	870 (280–1,200)	960 (400–1,120)
***Level of schooling***				
*n* (%) **none**	45 (4.00)	29 (5.4%)	6 (2.1%)	10 (3.3%)
*n* (%) **less than 8 y**	369 (32.7%)	218 (41.0%)	84 (29.2%)	67 (21.8%)
*n* (%) **8–11 y**	465 (41.3%)	192 (36.1%)	122 (42.4%)	151 (49.2%)
*n* (%) **finished high school**	159 (14.1%)	49 (9.2%)	52 (18.1%)	58 (18.9%)
*n* (%) **tertiary education**	89 (7.9%)	44 (8.3%)	24 (8.3%)	21 (6.8%)
*n* (%) **participant ever having been imprisoned**	159 (14.1%)	92 (17.2%)	39 (13.5%)	28 (9.1%)
*n* (%) **participant living in informal settlement** d	712 (63.1%)	304 (57.0%)	207 (71.9%)	201 (65.3%)
**Clinical (** ***n*** ** = 1,130)**				
*n* (%) **diabetes**	48 (4.3%)	36 (6.8%)	3 (1.1%)	9 (2.9%)
Median (IQR) **BMI**	24.4 (21.4–29.4)	22.7 (20.2–27.4)	25.1 (22.2–30.1)	26.5 (23.3–30.9)
Median (IQR) **current CD4 count (cells/µl)** e	NA	NA	434 (316–617)	403 (288–570)
*n* (%) **currently on ART** f	NA	NA	NA	120 (39.0%)
Median (IQR) **time on ART (y)**	NA	NA	NA	3.1 (1.1–6.5)
***Previous TB episode***				
*n* (%) **none**	845 (74.8%)	402 (75.3%)	247 (85.8%)	196 (63.6%)
*n* (%) **one**	247 (21.9%)	115 (21.5%)	37 (12.9%)	95 (30.8%)
*n* (%) **more than one**	38 (3.4%)	17 (3.2%)	4 (1.4%)	17 (5.5%)
*n* (%) **TB within the last 2 y**	133 (11.8%)	60 (11.2%)	15 (5.2%)	58 (18.8%)
*n* (%) **TB household contact**	240 (21.2%)	121 (22.7%)	63 (21.9%)	56 (18.2%)
***Symptoms***				
*n* (%) **cough**	707 (62.6%)	450 (84.3%)	124 (43.1%)	133 (43.2%)
*n* (%) **haemoptysis**	126 (11.2%)	94 (17.6%)	14 (4.9%)	18 (5.8%)
*n* (%) **fever**	119 (10.5%)	75 (14.0%)	21 (7.3%)	23 (7.47%)
*n* (%) **night sweats**	628 (55.6%)	395 (74.0%)	115 (39.9%)	118 (38.3%)
*n* (%) **weight loss**	458 (40.5%)	284 (53.2%)	78 (27.1%)	96 (31.2%)
*n* (%) **positive symptom screen**	838 (74.2%)	497 (93.1%)	160 (55.6%)	162 (52.6%)
**Health seeking behaviour (** ***n*** ** = 821)**				
*n* (%) **sought medical care**	170 (20.7%)	112 (23.0%)	19 (11.6%)	39 (23.1%)
*n* (%) **sputum sample sent by the clinic**	105 (61.8%)	69 (61.6%)	10 (52.6%)	26 (66.7%)
*n* (%) **CXR performed by the clinic**	54 (31.8%)	35 (31.3%)	4 (21.1%)	15 (38.5%)

a 3 missing values.

b 4 missing values.

c 2 missing values.

d 1 missing value.

e 30 missing values.

f 18 missing values.

BMI, body mass index; CXR, chest X-ray.

Median BMI was 24.4 (IQR 21.4–29.4). A quarter of individuals had a history of previous TB. The median CD4 count was 434 cells/µl (IQR 316–617) in newly diagnosed and 403 cells/µl (IQR 288–570) in known HIV-infected individuals. 40% of the known HIV-infected individuals were on antiretroviral therapy (ART) at the time of screening with a median time on ART of 3.1 y (IQR 1.1–6.5).

The most prevalent TB symptom among HIV negative TB suspects was cough (84.3%). A total of 160 (55.6%) of the newly diagnosed and 162 (52.6%) of the known HIV-infected individuals screened positive for TB symptoms. Only 20.7% (*n* = 170) of all individuals reporting symptoms had previously sought health care for their current symptoms. Of those who had sought health care, 61.8% had undergone sputum investigations and 31.8% had had chest radiography.

### Yield of TB Screening

Among all HIV-negative individuals (*n* = 5,551) or individuals with unknown HIV status (*n* = 85) who accessed the mobile service and were potentially eligible for TB screening, including those who were unable to provide a sputum sample, prevalence was 0.2% (95% CI 0.1–0.4) for smear-positive TB and 0.5% (95% CI 0.3–0.7) for culture-positive TB. Among individuals newly diagnosed with HIV, TB prevalence was 2.2% (95% CI 0.9–4.2) for smear-positive and 4.9% (95% CI 2.9–7.6) for culture-positive disease. Prevalence was 0.3% (95% CI 0–1.4) for smear-positive and 3.1% (95% CI 1.6–5.3) for culture-positive TB in individuals with known HIV infection.

The prevalence of smear-positive disease among individuals providing a sputum sample was 2.0% (95% CI 1.2–3.0) (Table 2). The prevalence of smear-positive TB was 2.2% (95% CI 1.1–4.0), 3.3% (95% CI 1.4–6.4), and 0.4% (95% CI 0–2.0) in HIV-negative TB suspects, those with newly diagnosed HIV infection, and those with known HIV infection, respectively. Prevalence of culture-positive TB was 5.5% (95% CI 4.2–7.1) overall. Median time to culture-positivity was 13.5 d (IQR 8–22). All isolates were sensitive to rifampicin and isoniazid.

**Table 2:** Tuberculosis prevalence (*n* = 1,011).

Variables	Total (*n* = 1,011)	HIV− (*n* = 491)	Newly Diagnosed HIV+ (*n* = 243)	Known HIV+ (*n* = 277)
***Smear result***				
***n*** ** (%) overall positive**	20 (2.0%)	11 (2.2%)	8 (3.3%)	1 (0.4%)
***n*** ** (%) scanty**	3 (0.3%)	1 (0.2%)	2 (0.8%)	0 (0.0%)
***n*** ** (%) 1+**	6 (0.6%)	3 (0.6%)	2 (0.8%)	1 (0.4%)
***n*** ** (%) 2+**	6 (0.6%)	4 (0.8%)	2 (0.8%)	0 (0.0%)
***n*** ** (%) 3+**	5 (0.5%)	3 (0.6%)	2 (0.8%)	0 (0.0%)
***Culture result***				
***n*** ** (%) negative**	746 (73.8%)	346 (70.5%)	180 (74.1%)	220 (79.4%)
***n*** ** (%) contaminated**	162 (16.0%)	88 (17.9%)	36 (14.8%)	38 (13.7%)
***n*** ** (%) MOTT**	47 (4.7%)	31 (6.3%)	9 (3.7%)	7 (2.5%)
***n*** ** (%) ** ***M. tuberculosis***	56 (5.5%)	26 (5.3%)	18 (7.4%)	12 (4.3%)
Median (IQR) **days to culture positivity**	13.5 (8–22)	12 (7–17)	13 (8–22)	19 (14.5–27)

MOTT, mycobateria other than TB.

TB prevalence was highest in HIV-infected patients with CD4 counts ≤200 cells/µl (18.6%; 95% CI 9.7–30.9), followed by patients with missing CD4 counts (7.7%; 95% CI 0.9–25.1). TB prevalence was 5.3% (95% CI 2.0–11.1), 4.3% (95% CI 1.6–9.1), and 2.8% (95% CI 0.9–6.3) in HIV-infected patients with CD4 counts of 201–350, 351–500, and >500 cells/µl, respectively.

### Contact Rates, Treatment Initiation, and Treatment Outcomes

A total of 56 individuals were diagnosed with TB. The baseline characteristics and symptoms are described in Table S3. Successful follow-up contact was made with 50 individuals (89.3%, 95% CI 78.1–96.0) (Table 3). Contact success was higher in those with smear-positive disease (95.0%, 95% CI 75.1–99.9) compared to those with smear-negative/culture-positive disease (86.1%, 95% CI 70.5–95.3). The median time to successful contact from time of positive result was 4 d (IQR 1–10) and was shorter for individuals with smear-positive TB (1 d, IQR 0–2). Successful contacting of patients with smear-positive disease rarely required more than one attempt, whereas contact of smear-negative cases involved one to two phone calls and often a home visit.

**Table 3:** Contact rates and treatment success in patients diagnosed with tuberculosis.

Variables	Total (*n* = 56)	Smear + (*n* = 20)	Smear −/Culture + (*n* = 36)
***n*** ** (%) successful contact**	50 (89.3%)	19 (95.0%)	31 (86.1%)
**Median (IQR) time to successful contact from availability of positive result (d)**	4 (1–10)	1 (0–2)	6 (4–20)
***Treatment started***			
***n*** ** (%) no**	2 (4.0%)	1 (5.3%)	1 (3.2%)
***n*** ** (%) unknown**	6 (12.0%)	1 (5.3%)	5 (16.1%)
***n*** ** (%) confirmed**	42 (84.0%)	17 (89.5%)	25 (80.7%)
**Median (IQR) time to treatment from screening date (d)**	27 (7–54)	6.5 (4.5–8)	45 (32–57)
**Median (IQR) time to treatment from availability of positive result (d)**	24 (3–50)	1.5 (0–6.5)	43 (29–74)
**Median (IQR) time to treatment from contact date (d)**	1 (0–8)	0.5 (0–25)	2 (0–23)
***Treatment outcome***			
***n*** ** (%) treatment completed or cured**	34 (81.0%)	12 (70.6%)	22 (88.0%)
***n*** ** (%) died**	2 (4.8%)	1 (5.9%)	1 (4.0%)
***n*** ** (%) defaulted**	5 (11.9%)	3 (17.6%)	2 (8.0%)
***n*** ** (%) treatment interruption**	1 (2.4%)	1 (5.9)	0 (0.0%)

Of the 50 individuals contacted, a total of 42 (84.0%) were confirmed to have started TB treatment. The median time between screening and treatment initiation was 27 d (IQR 7–54) overall, 6.5 d (IQR 4.5–8) for smear-positive and 45 d (IQR 32–57) for smear-negative/culture-positive cases. The median time between successful contact and treatment initiation was 1 d (IQR 0–8). Most individuals (30/42) who were initiated on treatment were initiated within 7 d following a successful contact. Thus the majority of individuals only needed one successful contact to link them to care.

The overall treatment success rate was 81.0% (*n* = 34/42) (95% CI 65.9–91.4). Two individuals died while on treatment, five defaulted, and one interrupted treatment, but restarted treatment 3 mo later.

**Figure 4:**
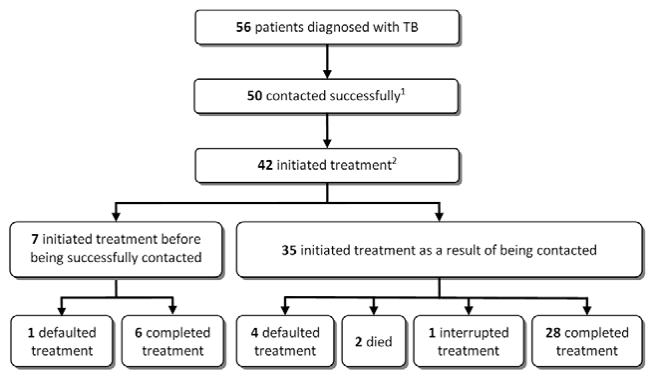
Losses between tuberculosis diagnosis to treatment completion. (1) The reasons for not being able to contact individuals were: relocation to an unknown area (*n* = 3), demolition of the area where the individual had lived (*n* = 2), and imprisonment (*n* = 1). (2) Two individuals had refused treatment and six individuals had not started treatment at their nearest clinic. Several attempts were made to contact these individuals, but all of them had moved to an unknown destination.

Of the 42 individuals who had initiated treatment, seven had started before a successful contact could be made (Figure 4). In these cases, the initiation of treatment was not triggered by the positive sputum result. These seven individuals had presented with their referral letter to a primary care clinic and were started on TB treatment on the basis of clinical presentation and investigations performed at the clinic.

### Costs

The incremental cost of actively screening 1,130 individuals for TB including treatment costs was US$83,559. US$22,367 (26.8%) were spent on laboratory tests, US$37,427 (44.8%) on staff salaries, and US$17,652 (21.1%) on TB treatment, with the remaining US$6,108 (7.3%) spent on transport, office rent and utilities, communication, and supplies (Table S1). The costs were US$1,117 per TB case detected and US$2,458 per TB case with a positive treatment outcome (cured or treatment completed).

Sensitivity analysis was performed for different levels of staff salaries and using discount rates for capital costs of 0% and 3%, assuming the same effectiveness. Substituting the clinical nurse practitioner with a staff nurse for all tasks would have reduced the cost per TB case diagnosed by 9.1% to US$1,015 and the cost per TB case with positive treatment outcome by 10.9% to US$2,190. Further reduction in costs would have been achieved if TB screening had been performed by a lay counsellor and follow-up of TB cases, supervision, and program management had been performed by a clinical nurse practitioner. The costs per TB case diagnosed would have been reduced to US$705 (36.9%) and the cost per TB case with a positive treatment outcome to US$1,681 (31.6%). Costs per TB case diagnosed increased by 4.9% to US$1,172 and by 5.1% US$1,174 assuming a discount factor of 0% and 3%, respectively. Changing the discount rate did not substantially affect the cost per TB case with positive treatment outcome.

## Discussion

This study demonstrated that community-based active TB case finding delivered via a mobile HIV testing service is feasible and has a high uptake and yield of diagnoses. It highlighted the losses between TB diagnosis and treatment completion. Outcomes of the 56 individuals diagnosed with TB were as follows: 34 completed treatment (60.7%), eight (14.3%) initiated treatment and defaulted or died, eight (14.3%) were contactable but did not initiate treatment, and six (10.7%) were not contacted. This study is unique in that it followed patients beyond the diagnosis of TB and ascertained treatment outcomes. Once a patient had started TB treatment, treatment success was more than 80%, which was as high as previously reported from public service clinics in the Western Cape [18]. Costs were US$1,117 per TB case diagnosed and US$2,458 per successfully treated TB case.

The service described in this study was accessed by a population with severe socio-economic deprivation as evidenced by high unemployment, low income, and low levels of education. Only one in five individuals with symptoms suggestive of TB had sought medical attention prior to accessing the service. More importantly only 13 of the 56 individuals diagnosed with TB had accessed medical care before attending the mobile clinic. It is well documented that TB patients and suspects present late to stationary health facilities, which contributes to delays in diagnosis, morbidity, and mortality [19].

The prevalence of HIV and severe immunodeficiency in this mobile HIV testing service were low compared to stationary services in the Western Cape [10]. This finding and the fact that only one sputum sample was examined explains the lower yield of screening in HIV-infected individuals in this study compared to studies screening individuals at stationary HIV testing sites [9],[20] and at time of ART eligibility screening [21]–[23]. Furthermore stationary clinics are likely to be accessed by sicker individuals with a higher probability of TB disease.

Interestingly, the overall prevalence of TB and the prevalence of smear-positive disease were lower in known compared to the newly diagnosed HIV-infected individuals. Median CD4 counts were comparable in both groups. Individuals known to be HIV positive regularly engage with the health care system and development of TB is therefore likely to be detected more rapidly. Several studies showed a benefit of screening HIV-positive individuals for TB regardless of symptoms [9]. These studies have been conducted among severely immune-suppressed individuals and might not be applicable to mobile services [20]–[23]. TB symptom screening regardless of HIV status is likely more appropriate for mobile settings. Using this strategy in this service would have decreased the cost per TB case detected by 15% to US$996, but two TB cases subsequently completing treatment would have been missed.

The success of active TB case finding in decreasing transmission relies on the treatment success of actively found cases. A study from Nepal reported higher treatment refusal and default rates in actively compared to passively detected cases [24]. This finding is of concern as it decreases the effectiveness of active TB case finding and promotes resistance. However, actively found cases in Nepal lived further away from the clinic compared to passively found cases resulting in reduced probability of being diagnosed passively and completing treatment. The results of our study are therefore encouraging in showing a treatment success rate of 80%, which is comparable to outcomes in passively detected cases reported from clinics in Cape Town [18]. The few other studies reporting treatment outcomes in actively found cases show similar results [25]–[28]. A total of 14 (25%) individuals diagnosed with TB in this study did not start treatment, which compares favourably to passively detected cases. Defaulters prior to treatment initiation are not part of the routine TB outcome reporting, but rates of initial defaulting of 17%–21% have been reported from stationary clinics in South Africa using passive case finding [29]–[32]. More recently a study conducted in Cape Town reported initial defaulting rates of patients with smear-negative/culture-positive TB as high as 39% in the context of passive case finding [33].

Treatment outcomes might be even more important than the yield of screening. A new rapid diagnostic, the Xpert MTB/RIF with an overall sensitivity of 90% in TB suspects reduced the time to start treatment from 56 to 5 d and dropout rates from 39% to 15% in smear-negative/culture-positive cases in a primary health care clinic in Cape Town [33]. The reduction in diagnostic delay is particularly important in mobile services. In our study contact success was higher in individuals with smear-positive disease, because results were available within 1–2 d, compared to individuals with smear-negative/culture-positive disease. Relatively more resources and time were spent contacting individuals with smear-negative/culture-positive disease, as patients' mobile phones had stopped working or had been lost, stolen, or passed on and individuals had moved to different locations in the meantime. Interventions to decrease losses along the screening pathway should be aimed at increased uptake and contact success. Possible interventions to increase uptake include providing entertainment such as films or incentives to complete screening. Contact success might be improved by providing incentives for individuals to return to receive their TB result. A similar approach has been shown to increase return for HIV results [34]. However, such interventions would increase the cost of the program and would have to be balanced against the potential benefit.

This study assessed the cost of community-based active TB case finding using a mobile screening unit. Three studies investigated the costs of active TB case finding in HIV-infected individuals only in stationary clinics in South Africa and Cambodia [23],[35],[36]. Prevalence of undiagnosed TB in these studies was 19%–26% [23],[36]; in part as a result, the cost per TB case diagnosed in 2011 US$ was more than three times lower (US$318–US$358) compared to our screening program [23],[35],[36]. None of these studies assessed treatment outcomes. The cost per TB case detected in the mobile screening unit was 4.7 times higher than a recent estimate for passive case finding in South Africa (US$251) [17]. However, the passive case-finding estimate assumed a smear positivity rate of 10%, which is 5 times higher than the one in this study. The unit cost per suspect screened is therefore roughly comparable.

It should be borne in mind however that active case finding is not an alternative to passive case detection (or its enhancement), but is additional to any existing active case-finding programme since it targets a different (often harder to reach) suspect population. A recent modelled cost-effectiveness analysis of passive case finding using smear microscopy, estimating a cost per case treated (in 2011 US$) of US$812 for smear only and US$939 for smear plus culture, found a cost per disability-adjusted life-year (DALY) averted of US$120 and US$457, respectively [37]. This estimate of cost per DALY averted are clearly well below a willingness to pay threshold of one gross domestic product (GDP) per capita per DALY averted [38] (over US$7280 for South Africa [39]). In this context, our cost of US$2,458 per case treated, although 3-fold higher than the cost per case treated under passive case detection, suggests that further investigation into the cost-effectiveness of active case finding in South Africa is warranted.

Mobile HIV testing services are widespread in sub-Saharan Africa and thus these results are likely to inform policy makers when considering adding active TB case finding to existing mobile services in a similarly incremental manner. The study was conducted as part of a routine service and provides an opportunity to understand the challenges faced by mobile services. Mobile services operate under time, space, and weather constraints. As a result a considerable number of individuals were not referred or did not want to wait for TB screening. Furthermore the population accessing mobile services are healthier, less health care seeking, and more mobile than individuals accessing stationary services, resulting in reduced yield, contact, and treatment initiation rates.

A limitation of the study is that it was conducted at a single study site; the findings can therefore only be generalised to similar settings with comparable levels of deprivation and TB prevalence. In addition generalisability to countries outside of sub-Saharan Africa with low HIV prevalence is limited. TB prevalence surveys report similar rates of undiagnosed TB across sub-Saharan Africa including surveys from Cape Town [40]–[48]. While this might suggest a similar yield of active TB case finding in other sub-Saharan African settings, infrastructure and resources are very different in South Africa compared to other African countries affected by the HIV-TB epidemic. Thus liquid culture and sputum induction might not be viable in these settings. However, alternative strategies such as collection of spot sputum samples, use of a mechanical nebuliser [49], investigations limited to smear microscopy only [6] and Xpert MTB/RIF might be more feasible in these settings.

South Africa has the second highest absolute number of TB cases in the world and, together with other countries, including Swaziland, Lesotho, and Botswana, in the sub-region has the highest rates of TB globally [50]. Importantly, this epidemic has completely failed to be controlled by the WHO DOTS strategy and other more intensive interventions are needed such as active case finding [51]. As such, these study findings are relevant and very important to a critical geographic region worst affected by TB. However, further research of mobile integrated chronic and infectious disease screening in other sub-Saharan African settings is needed to address this question.

A recent study from Kenya concluded that the highest impact would be achieved when population-based active TB case finding was combined with universal HIV testing and improved diagnosis of smear-negative TB [52]. Our active TB case-finding program provided such integrated TB and HIV services combined with improved TB diagnostics. Our study shows that population-based integrated disease screening for TB and HIV has a high uptake, yield, and treatment success at reasonable costs. The study highlights the challenges faced by mobile population-based services including high contamination rates, time and weather constraints, as well as problems contacting individuals with positive results. New and pragmatic approaches including new diagnostics such as the Xpert MTB/RIF, information technology, incentives, and targeting of high risk groups are needed to improve efficiency, reduce costs, and expand population-based integrated screening services.

## Supporting Information

Table S1 **Details of cost data collection.** Cost inputs. (1) Includes tents, chairs, nebuliser, masks, tubing, disinfectant, stationery; (2) Includes smear microscopy, liquid culture, drug-susceptibility testing, line probe assay.
(DOCX)

Table S2 **Results from the time-and-motion study.** Time-and-motion studies at the mobile unit were conducted over one week in August 2010 and two wk in January 2011, with a total of 13 complete screening days being observed. There was no difference in the number of patients screened per day and time allocated to different tasks during the 2 screening periods. However, the number of days per months when TB screening was conducted was higher in summer compared to winter months.
(DOCX)

Table S3 **Socio-demographic, clinical characteristics, and health seeking behaviour among TB cases.** (1) 1 missing value; (2) 1 missing value.
(DOCX)

## Abbreviations

ART: antiretroviral therapy
DALY: disability-adjusted life-year
IQR: interquartile range
TB: tuberculosis
WHO: World Health Organization

## References

[1] WHO Three I's Meeting (2008) Geneva: World Health Organization. Available: http://www.who.int/hiv/pub/meetingreports/WHO_3Is_meeting_report.pdf. Accessed 31 August 2011.

[2] WHO (2010) Guidelines for intensified tuberculosis case-finding and isoniazid preventive therapy for people living with HIV in resource-constrained settings. Geneva: World Health Organization. Available: http://www.who.int/hiv/pub/tb/9789241500708/en/index.html. Accessed 20 February 2011.

[3] International Union Against Tuberculosis and Lung Disease (2007) Best practice for the care of patients with tuberculosis: a guide for low-income countries. Paris: International Union Against Tuberculosis and Lung Disease. Available: http://www.theunion.org/index.php/en/resources/scientific-publications/tuberculosis/item/104-best-practice-for-the-care-of-patients-with-tuberculosis-a-guide-for-low-income-countries. Accessed 21/9/2011.

[4] International Union Against Tuberculosis and Lung Disease (2002) Interventions for TB control and elimination. Paris: International Union Against Tuberculosis and Lung Disease. Available: http://www.tbrieder.org/publications/books_english/interventions.pdf. Accessed 21/9/2011.

[5] WHO (2006) Guidance for national tuberculosis programmes on the management of tuberculosis in children. Geneva: World Health Organization. Available: http://whqlibdoc.who.int/hq/2006/WHO_HTM_TB_2006.371_eng.pdf. Accessed 21/9/2011. 24999516

[6] Corbett EL, Bandason T, Duong T, Dauya E, Makamure B, et al (2010) Comparison of two active case-finding strategies for community-based diagnosis of symptomatic smear-positive tuberculosis and control of infectious tuberculosis in Harare, Zimbabwe (DETECTB): a cluster-randomised trial. Lancet 376: 1244–1253. 20923715 10.1016/S0140-6736(10)61425-0 PMC2956882

[7] WHO (2011) Scoping meeting for the development of guidelines on screening for active TB. Geneva: World Health Organization. Available: http://www.who.int/tb/TBscreeningmeetingreport2011.pdf. Accessed 31 August 2011.

[8] Golub JE, Mohan CI, Comstock GW, Chaisson RE (2005) Active case finding of tuberculosis: historical perspective and future prospects. Int J Tuberc Lung Dis 9: 1183–1203. 16333924 PMC4472641

[9] Kranzer K, Houben RM, Glynn JR, Bekker LG, Wood R, et al (2010) Yield of HIV-associated tuberculosis during intensified case finding in resource-limited settings: a systematic review and meta-analysis. Lancet Infect Dis 10: 93–102. 20113978 10.1016/S1473-3099(09)70326-3 PMC3136203

[10] Van Schaik N, Kranzer K, Wood R, Bekker LG (2010) Earlier HIV diagnosis - are mobile services the answer? S Afr Med J 100: 671–674. 21080998 10.7196/samj.4162

[11] Western Cape Department of Health.The Western Cape Antiretroviral Programme (2006) Cape Town: Provincial Government of the Western Cape: Western Cape Department of Health. Available: http://web.uct.ac.za/depts/epi/artrollout/. Accessed 2 February 2011.

[12] WHO (2000) Costing guidelines for HIV/AIDS prevention strategies. UNAIDS Best Practice Collection - Key Materials. Geneva: UNAIDS. Available: http://www.unaids.org/html/pub/publications/irc-pub05/jc412-costguidel_en_pdf.pdf. Accessed 14 September 2011.

[13] Drummond MF, O'Brien B, Stoddart GL (1997) Methods for the economic evaluation of health care programmes. New York: Oxford University Press.

[14] Kumaranayake L (2000) The real and the nominal? Making inflationary adjustments to cost and other economic data. Health Policy Plan 15: 230–234. 10837047 10.1093/heapol/15.2.230

[15] International Monentary Fund. Available: http://www.imf.org. Accessed 15 Augut 2011.

[16] Sinanovic E, Floyd K, Dudley L, Azevedo V, Grant R, et al (2003) Cost and cost-effectiveness of community-based care for tuberculosis in Cape Town, South Africa. Int J Tuberc Lung Dis 7: S56–62. 12971655

[17] University of the Witswatersrand and Centre for Global Health and Development, Boston University (20110 The cost of the Xpert diagnostic algorithm for TB. Results of the national TB cost model (NTCM) 2011/12 to 2016/17. Witswatersrand and Boston: University of the Witswatersrand and Centre for Global Health and Development, Boston University.

[18] Final evaluation report enhanced tuberculosis adherence programm. Cape Town. Available: http://www.mrc.ac.za/healthsystems/finalr.pdf. Accessed 1 September 2011.

[19] Storla DG, Yimer S, Bjune GA (2008) A systematic review of delay in the diagnosis and treatment of tuberculosis. BMC Public Health 8: 15. 18194573 10.1186/1471-2458-8-15 PMC2265684

[20] Munseri PJ, Bakari M, Pallangyo K, Sandstrom E (2010) Tuberculosis in HIV voluntary counselling and testing centres in Dar es Salaam, Tanzania. Scand J Infect Dis 42: 767–774. 20586671 10.3109/00365548.2010.495725

[21] Lawn SD, Edwards DJ, Kranzer K, Vogt M, Bekker LG, et al (2009) Urine lipoarabinomannan assay for tuberculosis screening before antiretroviral therapy diagnostic yield and association with immune reconstitution disease. AIDS 23: 1875–1880. 20108382 10.1097/qad.0b013e32832e05c8

[22] Lawn SD, Brooks SV, Kranzer K, Nicol MP, Whitelaw A, et al (2011) Screening for HIV-associated tuberculosis and rifampicin resistance before antiretroviral therapy using the Xpert MTB/RIf assay: a prospective study. PLoS Med 8: e1001067 doi:10.1371/journal.pmed.1001067. 21818180 10.1371/journal.pmed.1001067 PMC3144215

[23] Bassett IV, Wang B, Chetty S, Giddy J, Losina E, et al (2010) Intensive tuberculosis screening for HIV-infected patients starting antiretroviral therapy in Durban, South Africa. Clin Infect Dis 51: 823–829. 20735240 10.1086/656282 PMC3204934

[24] Cassels A, Heineman E, LeClerq S, Gurung PK, Rahut CB (1982) Tuberculosis case-finding in Eastern Nepal. Tubercle 63: 175–185. 7179484 10.1016/s0041-3879(82)80028-7

[25] den Boon S, Verver S, Lombard CJ, Bateman ED, Irusen EM, et al (2008) Comparison of symptoms and treatment outcomes between actively and passively detected tuberculosis cases: the additional value of active case finding. Epidemiol Infect 136: 1342–1349. 18177518 10.1017/S0950268807000106 PMC2870736

[26] Santha T, Renu G, Frieden TR, Subramani R, Gopi PG, et al (2003) Are community surveys to detect tuberculosis in high prevalence areas useful? Results of a comparative study from Tiruvallur District, South India. Int J Tuberc Lung Dis 7: 258–265. 12661841

[27] Gupta A, Nayak U, Ram M, Bhosale R, Patil S, et al (2007) Postpartum tuberculosis incidence and mortality among HIV-infected women and their infants in Pune, India, 2002–2005. Clin Infect Dis 45: 241–249. 17578786 10.1086/518974

[28] Harper I, Fryatt R, White A (1996) Tuberculosis case finding in remote mountainous areas–are microscopy camps of any value? Experience from Nepal. Tuber Lung Dis 77: 384–388. 8796258 10.1016/s0962-8479(96)90107-0

[29] Botha E, den Boon S, Lawrence KA, Reuter H, Verver S, et al (2008) From suspect to patient: tuberculosis diagnosis and treatment initiation in health facilities in South Africa. Int J Tuberc Lung Dis 12: 936–941. 18647454

[30] Botha E, Den Boon S, Verver S, Dunbar R, Lawrence KA, et al (2008) Initial default from tuberculosis treatment: how often does it happen and what are the reasons? Int J Tuberc Lung Dis 12: 820–823. 18544210

[31] Edginton ME, Wong ML, Phofa R, Mahlaba D, Hodkinson HJ (2005) Tuberculosis at Chris Hani Baragwanath Hospital: numbers of patients diagnosed and outcomes of referrals to district clinics. Int J Tuberc Lung Dis 9: 398–402. 15830744

[32] Dunbar R, Lawrence K, Verver S, Enarson DA, Lombard C, et al (2011) Accuracy and completeness of recording of confirmed tuberculosis in two South African communities. Int J Tuberc Lung Dis 15: 337–343. 21333100

[33] Boehme CC, Nicol MP, Nabeta P, Michael JS, Gotuzzo E, et al (2011) Feasibility, diagnostic accuracy, and effectiveness of decentralised use of the Xpert MTB/RIF test for diagnosis of tuberculosis and multidrug resistance: a multicentre implementation study. Lancet 377: 1495–1505. 21507477 10.1016/S0140-6736(11)60438-8 PMC3085933

[34] Thornton R (2005) The impact of incentives on learning HIV status: evidence from a field experiment. Cambridge (Massachusetts): Havard University.

[35] Hausler HP, Sinanovic E, Kumaranayake L, Naidoo P, Schoeman H, et al (2006) Costs of measures to control tuberculosis/HIV in public primary care facilities in Cape Town, South Africa. Bull World Health Organ 84: 528–536. 16878226 10.2471/blt.04.018606 PMC2627402

[36] Sutton BS, Arias MS, Chheng P, Eang MT, Kimerling ME (2009) The cost of intensified case finding and isoniazid preventive therapy for HIV-infected patients in Battambang, Cambodia. Int J Tuberc Lung Dis 13: 713–718. 19460246

[37] Dowdy DW, O'Brien MA, Bishai D (2008) Cost-effectiveness of novel diagnostic tools for the diagnosis of tuberculosis. Int J Tuberc Lung Dis 12: 1021–1029. 18713499

[38] Shillcutt SD, Walker DG, Goodman CA, Mills AJ (2009) Cost effectiveness in low- and middle-income countries: a review of the debates surrounding decision rules. Pharmacoeconomics 27: 903–917. 19888791 10.2165/10899580-000000000-00000 PMC2810517

[39] World Bank (2012) GDP per capita (current US$). Available: http://data.worldbank.org/indicator/NY.GDP.PCAP.CD. Accessed 2 May 2012.

[40] Ayles H, Schaap A, Nota A, Sismanidis C, Tembwe R, et al (2009) Prevalence of tuberculosis, HIV and respiratory symptoms in two Zambian communities: implications for tuberculosis control in the era of HIV. PLoS One 4: e5602 doi:10.1371/journal.pone.0005602. 19440346 10.1371/journal.pone.0005602 PMC2680044

[41] Middelkoop K, Bekker LG, Myer L, Whitelaw A, Grant A, et al (2010) Antiretroviral program associated with reduction in untreated prevalent tuberculosis in a South African township. Am J Respir Crit Care Med 182: 1080–1085. 20558626 10.1164/rccm.201004-0598OC PMC2970849

[42] Corbett EL, Bandason T, Cheung YB, Makamure B, Dauya E, et al (2009) Prevalent infectious tuberculosis in Harare, Zimbabwe: burden, risk factors and implications for control. Int J Tuberc Lung Dis 13: 1231–1237. 19793427 PMC3374846

[43] Wood R, Middelkoop K, Myer L, Grant AD, Whitelaw A, et al (2007) Undiagnosed tuberculosis in a community with high HIV prevalence: implications for tuberculosis control. Am J Respir Crit Care Med 175: 87–93. 16973982 10.1164/rccm.200606-759OC PMC1899262

[44] Demissie M, Zenebere B, Berhane Y, Lindtjorn B (2002) A rapid survey to determine the prevalence of smear-positive tuberculosis in Addis Ababa. Int J Tuberc Lung Dis 6: 580–584. 12102296

[45] Sekandi JN, Neuhauser D, Smyth K, Whalen CC (2009) Active case finding of undetected tuberculosis among chronic coughers in a slum setting in Kampala, Uganda. Int J Tuberc Lung Dis 13: 508–513. 19335958 PMC2842997

[46] Guwatudde D, Zalwango S, Kamya MR, Debanne SM, Diaz MI, et al (2003) Burden of tuberculosis in Kampala, Uganda. Bull World Health Organ 81: 799–805. 14758406 PMC2572356

[47] den Boon S, van Lill SW, Borgdorff MW, Enarson DA, Verver S, et al (2007) High prevalence of tuberculosis in previously treated patients, Cape Town, South Africa. Emerg Infect Dis 13: 1189–1194. 17953090 10.3201/eid1308.051327 PMC2828063

[48] Pronyk PM, Joshi B, Hargreaves JR, Madonsela T, Collinson MA, et al (2001) Active case finding: understanding the burden of tuberculosis in rural South Africa. Int J Tuberc Lung Dis 5: 611–618. 11467367

[49] Kranzer K, Olson L, van Schaik N, Raditlhalo E, Hudson E, et al (2011) Quality of induced sputum using a human-powered nebuliser in a mobile human immunodeficiency virus testing service in South Africa. Int J Tuberc Lung Dis 15: 1077–1081. 21740671 10.5588/ijtld.10.0684

[50] WHO (2011) Global tuberculosis control. Geneva: World Health Organization. Available: http://whqlibdoc.who.int/publications/2011/9789241564380_eng.pdf.

[51] Harries AD, Zachariah R, Corbett EL, Lawn SD, Santos-Filho ET, et al (2010) The HIV-associated tuberculosis epidemic–when will we act? Lancet 375: 1906–1919. 20488516 10.1016/S0140-6736(10)60409-6

[52] van't Hoog AH, Laserson KF, Githui WA, Meme HK, Agaya JA, et al (2011) High prevalence of pulmonary tuberculosis and inadequate case finding in rural western Kenya. Am J Respir Crit Care Med 183: 1245–1253. 21239690 10.1164/rccm.201008-1269OC

