# Supplementary material for: Feasibility, Yield, and Cost of Active Tuberculosis Case Finding Linked to a Mobile HIV Service in Cape Town, South Africa: A Cross-sectional Study
Source: PLoS Med. 2012 Aug 7;9(8):e1001281. doi: 10.1371/journal.pmed.1001281 (PMC3413719; doi:10.1371/journal.pmed.1001281)
Supplement: Table S1 — Details of cost data collection. Cost inputs. (1) Includes tents, chairs, nebuliser, masks, tubing, disinfectant, stationery; (2) Includes smear microscopy, liquid culture, drug-susceptibility testing, line probe assay. (DOCX) [file pmed.1001281.s001.docx]

**Table S1**

| **Cost item** | **Proportional allocation to the active case-finding project** | **Data source** | **Costs in 2011 USD** |
| --- | --- | --- | --- |
| ***Capital costs*** |  |  |  |
| **Generator** | 52% | Own data | 263 |
| **Training** | 100% | Own data | 2075 |
| ***Recurrent costs*** |  | Own data |  |
| **Staff** |  |  |  |
| **Clinical nurse specialist** | 41% (69% screening, 27% follow-up, 4% training) | Own data | 27,048 |
| **Lay counsellor** | 5% |  | 643 |
| **Project Manager** | 7% |  | 7663 |
| **Transport** | 13% | Own data | 1284 |
| **Office rent and utilities** | 10% | Own data | 1452 |
| **Equipment and consumables^1^** | 100% | Own data | 3111 |
| **Laboratory tests^2^** | 100% | Own data | 22,367 |
| **TB treatment** | 100% | Literature | 17,652 |
| ***Total costs*** |  |  | 83,559 |
