# Supplementary material for: Feasibility, Yield, and Cost of Active Tuberculosis Case Finding Linked to a Mobile HIV Service in Cape Town, South Africa: A Cross-sectional Study
Source: PLoS Med. 2012 Aug 7;9(8):e1001281. doi: 10.1371/journal.pmed.1001281 (PMC3413719; doi:10.1371/journal.pmed.1001281)
Supplement: Table S2 — Results from the time-and-motion study. Time-and-motion studies at the mobile unit were conducted over one week in August 2010 and two wk in January 2011, with a total of 13 complete screening days being observed. There was no difference in the number of patients screened per day and time allocated to different tasks during the 2 screening periods. However, the number of days per months when TB screening was conducted was higher in summer compared to winter months. (DOCX) [file pmed.1001281.s002.docx]

**Table S2**

| **Variable** | **Median (interquartile range)** |
| --- | --- |
| **Number of patients screened per day** | 8 (4-10) |
| ***Total staff time per task per day*** |  |
| **Patient-time per patient (min)** | 10 (9-11) |
| **Packing and decontamination of equipment (min)** | 97 (81-130) |
| **Driving (min)** | 73 (46-95) |
| **Break (min)** | 90 (38-120) |
| **Total time (min)** | 545 (485-570) |
