# Supplementary material for: Feasibility, Yield, and Cost of Active Tuberculosis Case Finding Linked to a Mobile HIV Service in Cape Town, South Africa: A Cross-sectional Study
Source: PLoS Med. 2012 Aug 7;9(8):e1001281. doi: 10.1371/journal.pmed.1001281 (PMC3413719; doi:10.1371/journal.pmed.1001281)
Supplement: Table S3 — Socio-demographic, clinical characteristics, and health seeking behaviour among TB cases. (1) 1 missing value; (2) 1 missing value. (DOCX) [file pmed.1001281.s003.docx]

| **Variables** |  | **Total**  **N=56** | **HIV-**  **N=26** | **Newly diagnosed HIV+**  **N=18** | | **Known HIV+**  **N=12** |
| --- | --- | --- | --- | --- | --- | --- |
| **Socio-demographic** |  |  |  | |  |  |
| **Age (years)** | Median (IQR) | 38.7 (27.6-44.7) | 39.1 (27.5-46.5) | | 34.7 (29.2-43.7) | 39.7 (29.4-44.3) |
| **Male gender** | N (%) | 27 (48.2%) | 17 (65.4%) | | 7 (38.9%) | 3 (25.0%) |
| ***Smoking*** |  |  |  | |  |  |
| **Never** | N (%) | 27 (48.2%) | 8 (30.8%) | | 11 (61.1%) | 8 (66.7%) |
| **Stopped** | N (%) | 1 (1.8%) | 0 (0%) | | 0 (0%) | 1 (8.3%) |
| **Currently** | N (%) | 28 (50.0%) | 18 (69.2%) | | 7 (38.9%) | 3 (25.0%) |
| ***Alcohol consumption^1^*** |  |  |  | |  |  |
| **Never** | N (%) | 27 (49.1%) | 12 (48.0%) | | 10 (55.6%) | 5 (41.7%) |
| **Once per week** | N (%) | 12 (21.8%) | 7 (28.0%) | | 3 (16.7%) | 2 (16.7%) |
| **2-3 time per week** | N (%) | 11 (20.0%) | 3 (12.0%) | | 5 (27.8%) | 3 (25.0%) |
| **Every day** | N (%) | 5 (9.1%) | 3 (12.0%) | | 0 (0%) | 2 (16.7%) |
| ***Current relationship^3^*** |  |  |  | |  |  |
| **Single** | N (%) | 38 (67.9%) | 16 (61.5%) | | 13 (72.2%) | 9 (75.0%) |
| **Partner** | N (%) | 16 (28.6%) | 9 (34.6%) | | 5 (27.8%) | 2 (16.7%) |
| **Divorced** | N (%) | 2 (3.6%) | 1 (3.9%) | | 0 (0%) | 1 (8,3%) |
| **Regular income** | N (%) | 49 (87.5%) | 21 (80.8%) | | 17 (94.4%) | 11 (91.7%) |
| ***Source of income*** |  |  |  | |  |  |
| **Government grants** | N (%) | 11 (22.4%) | 4 (19.1%) | | 3 (17.7%) | 4 (36.3%) |
| **Casual work** | N (%) | 16 (32.7%) | 6 (28.6%) | | 8 (47.1%) | 2 (18.2%) |
| **Regular work** | N (%) | 22 (44.9%) | 11 (52.4%) | | 6 (35.3%) | 5 (45.5%) |
| **Income per month (ZAR)** | Median (IQR) | 1000 (500-1216) | 1010 (600-1200) | | 800 (240-1300) | 1080 (700-2200) |
|  |  |  |  | |  | *Continued…* |
| *Continued: Appendix 3: Socio-demographic, clinical characteristics and health seeking behaviour among TB cases* | | | | | | |
| **Variables** |  | **Total**  **N=56** | **HIV-**  **N=26** | **Newly diagnosed HIV+**  **N=18** | | **Known HIV+**  **N=12** |
| ***Level of schooling^2^*** |  |  |  | |  |  |
| **None** | N (%) | 2 (3.6%) | 1 (4.0%) | | 1 (5.6%) | 0 (0%) |
| **Less than 8 years** | N (%) | 24 (43.6%) | 12 (48.0%) | | 7 (38.9%) | 5 (41.7%) |
| **8-11 years** | N (%) | 22 (40.0%) | 9 (36.0%) | | 9 (50.0%) | 4 (33.3%) |
| **Finished high school** | N (%) | 3 (5.4%) | 1 (4.0%) | | 0 (0%) | 2 (16.7%) |
| **Tertiary education** | N (%) | 4 (7.3%) | 2 (8.0%) | | 1 (5.6%) | 1 (8.3%) |
| **Participant ever having been imprisoned** | N (%) | 14 (25.0%) | 10 (38.5%) | | 4 (22.2%) | 0 (0%) |
| **Participant living in informal settlement^6^** | N (%) | 38 (67.9%) | 18 (69.2%) | | 13 (72.2%) | 7 (58.3%) |
| **Clinical** |  |  |  | |  |  |
| **Diabetes** | N (%) | 2 (3.6%) | 0 (0%) | | 0 (0%) | 2 (16.7%) |
| **BMI** | Median (IQR) | 22.3 (19.8-25.6) | 21 (20.2-25.8) | | 22.6 (19.4-23.9) | 24.1 (20.7-25.7) |
| **Current CD4 count (cells/µl)^7^** | Median (IQR) |  |  | | 283.5 (129.5-443) | 285.5 (154-427.5) |
| **Currently on ART** | N (%) |  |  | |  | 3 (25.0%) |
| **No past TB history** | N (%) | 43 (76.8%) | 21 (80.8%) | | 14 (77.8%) | 8 (66.7%) |
| **TB household contact** | N (%) | 9 (16.1%) | 3 (11.5%) | | 1 (5.6%) | 5 (41.7%) |
| ***Symptoms*** |  |  |  | |  |  |
| **Cough > 2 weeks** | N (%) | 38 (67.9%) | 21 (80.8%) | | 9 (50.0%) | 4 (33.3%) |
| **Cough** | N (%) | 44 (78.6%) | 23 (88.5%) | | 12 (66.7%) | 9 (75.0%) |
| **Haemoptysis** | N (%) | 10 (17.9%) | 6 (23.1%) | | 3 (16.7%) | 1 (8.3%) |
| **Fever** | N (%) | 6 (10.7%) | 3 (11.5%) | | 1 (5.6%) | 2 (16.7%) |
| **Night sweats** | N (%) | 44 (78.6%) | 24 (92.3%) | | 10 (55.6%) | 10 (83.3%) |
| **Weight loss** | N (%) | 37 (66.1%) | 20 (76.9%) | | 10 (55.6%) | 7 (58.3%) |
| **Health seeking behaviour (N=50)** |  |  |  | |  |  |
| **Sought medical care** | N (%) | 13 (26.0%) | 6 (24.0%) | | 4 (28.6%) | 3 (27.3%) |
| **Sputum sample sent by the clinic** | N (%) | 8 (61.5%) | 4 (66.7%) | | 2 (50.0%) | 2 (66.7%) |
| **CXR performed by the clinic** | N (%) | 3 (23.1%) | 0 (0%) | | 1 (25.0%) | 2 (66.7%) |
